# Gynecological complications in long-term survivors after allogeneic hematopoietic cell transplantation—a single-center real-life cross-sectional study

**DOI:** 10.3389/fmed.2022.956867

**Published:** 2022-09-15

**Authors:** Huina Su, Xinyu Zhou, Yanli Zhao, Yue Lu, DeYan Liu, Janping Zhang, Xin Yang

**Affiliations:** ^1^Department of Obstetrics and Gynecology, Peking University People's Hospital, Beijing, China; ^2^Department of Obstetrics and Gynecology, Beijing Tsinghua Changgung Hospital, Beijing, China; ^3^Department of Bone Marrow Transplantation, Hebei Yanda Lu Daopei Hospital, Langfang, China

**Keywords:** hematopoietic stem cell transplantation, premature ovarian insufficiency, conditioning regimen, myeloablative conditioning regimen, obstetrics and gynecology

## Abstract

**Background and objectives:**

Hematopoietic stem cell transplantation (HCT) is a treatment for hematopoietic diseases. However, most cured female patients may suffer from premature ovarian insufficiency (POI) after HCT, which is mainly caused by the pre-HCT conditioning regimen. Hence, this study aims to explore the impact of HCT treatment on reproductive and ovarian functions in female survivors.

**Methods:**

A total of 55 female participants under the age of 40, who underwent HCT and met the inclusion criteria were enrolled. Data related to blood disease, menstruation, and fertility in the 3 years following HCT were collected.

**Results:**

The involved patients received transplantation at different age stages, ranging from 8 to 37. All patients, except those with aplastic anemia (AA; 5/55), received a myeloablative conditioning regimen, usually modified total body irradiation/cyclophosphamide (TBI/Cy; 25/55) or modified Busulfan/cyclophosphamide (Bu/Cy; 23/55). Among women (42/55) who menstruated before HCT, 16.67% (7/42) had a spontaneous menstrual relapse and 83.3% (35/42) had amenorrhea after HCT. 72.7% (40/55) could be regarded as having POI. This proportion included 100% (25/25) of women aged 21–40 at the time of HCT, 62.5% (15/24) of those aged 11–20, and 0% (0/6) of those ≤10 years old. Patients with AML were more likely to have POI (95.7%). Patients aged ≤10 years (0%) or 11–20 years (16.7%) at the time of HCT were less likely to have moderate to severe menopause than those 21–40 years old (44%).

**Conclusion:**

The prevalence of POI following HCT was high and POI was associated with age, conditioning regimen, and type of blood disease.

## 1. Introduction

Hematopoietic stem cell transplantation (HCT) is an established treatment for many congenital or acquired disorders of the hematopoietic system and some other life-threatening diseases (1–5). More than 50,000 patients worldwide receive the treatment annually, including children, adolescents, and women of childbearing age. In China, the annual number of transplants reached more than 10,000 for the first time in 2019. The number of patients with pediatric (≤ 18 years of age) was 12.79%. The number of haploidentical donor (HID) HCT first exceeded 5,000 per year, which is much higher than that in the US or Europe (1,769 and 3,538 [10] in 2019) (6, 7). The group from Peking University established and enriched the Beijing Protocol which makes up 94% of HID HCTs in China. Further studies have shown that the “Beijing Protocol' in HID can provide comparable outcomes to matched sibling donors (MSD) or unrelated donor (URD) HCT in both benign diseases and hematologic malignancies (8). Over the last decades, survival rates of childhood, adolescent, and young adulthood (CAYA) cancer have remarkably increased thanks to substantial improvements in the comprehension of cancer molecular biology, refinement of diagnostic techniques, and novel treatment strategies (9–11). Hypogonadism secondary to antineoplastic treatment is called hypergonadotropic hypogonadism (characterized by elevated levels of luteinizing hormones and FSH owing to the lack of negative feedback from the gonads) (12). According to multiple published analyses, the incidence of ovarian failure ranges from 44 to 100% among transplant recipients during childhood, with clinical and demographical heterogeneity of different study cohorts accounting for most of this variability (13–15).

Cancers occurring in childhood and adolescence differ markedly from cancers in adults in their incidence and tumor characteristics. Worldwide, the average annual incidence in children aged less than 15 years is 140 new cases per million children, although there are three-fold variations between world regions and ethnic groups. The most common cancers in children are leukemia and lymphoma, while the major cancers among adults, such as carcinoma of the lung, breast, or colon, are rare in children. Cancer treatments are improving, but they are also often reproductive toxicity, leading an increasing number of young cancer survivors to seek personalized fertility preservation strategies (16, 17). The loss of fertility can negatively impact the quality of life (QOL) of young cancer survivors (18, 19), and women diagnosed with cancer show that the ability to have children in the future is very important (20). In fact, among young women diagnosed with cancer, the potential loss of fertility can sometimes be more stressful than the cancer diagnosis itself (21). The American Society of Clinical Oncology recommends that, as part of pre-cancer treatment education and informed consent, healthcare providers address infertility risks in patients treated during their reproductive years and be prepared to discuss fertility preservation options and/or refer all patients to fertility specialists (22). These referrals are essential because studies have shown that receiving counseling for precancer treatment regarding fertility preservation significantly improves QOL scores after cancer treatment in women of childbearing age (23). In addition, counseling with a fertility specialist and subsequent attempts to preserve fertility were associated with increased quality of life compared to women who only received counseling from an oncologist (23).

The myeloablative regimen conditioning (MAC) mBuCy regimen in MSD-HCT and the mBuCy+ATG regimen in haplo-HCT are the most popular in China and achieve remarkable results. Reduced-intensity conditioning (RIC) or intensified conditioning regimen is also used for subgroups of patients. High-dose radiotherapy and chemotherapy, especially the TBI and alkylating agents involved in the MAC cause damage to oocytes, granulosa cells, and ovarian stroma resulting in higher rates of POI. In detail, when the conditioning regimen administered in adult women includes total body irradiation (TBI), gonadal failure is extremely frequent and affects almost 100% of the patients for exposures above 10 Gy (24–26). The incidence of POI in hematological patients receiving conventional chemotherapy before HCT is 65–86% (27), rising to close to 100% following MAC, giving a probability of future pregnancy of less than 1% in the latter group (28). It has been established that factors affecting reproduction and ovarian function after HCT include the type of conditioning regimen, age, and pubertal status at the time of HCT, type of HCT, and types of hematologic disorders. In addition, osteoporosis, cardiovascular, neurological, and genitourinary tract diseases are common long-term risks that contribute to mortality among patients with POI (29).

In 2019, the total number of HCTs in China reached more than 10,000 for the first time benefiting from the Beijing Protocol. Although some studies have been conducted around the world, there is little information regarding the impact of HCT on reproduction and ovarian function in Chinese women. Protective measures and hormone replacement therapy post-HCT have not been fully investigated. To the best of our knowledge, this paper would be the first retrospective and prospective study on reproduction and ovarian function in Chinese women following allogeneic HCT. An exploration of the factors contributing to ovarian damage is expected to give insights into future protective practices.

## 2. Methods

### 2.1. Study design and procedure

The present study consisted of data analysis of a cross-sectional study of baseline data collected by questionnaires. First of all, we obtained the information of all female patients who visited the Hebei Yanda Ludaopei Hospital between 1 January and 31 December 2017 through the case database, and then obtained the patient's informed consent through outpatient or telephone or WeChat. Briefly, patients after HCT with the hematopoietic disease were recruited from outpatient hematology clinics. A data collection form was utilized to collect study-related information which included age at HCT, height, weight, disease type, lines of chemotherapy, conditioning regimen, menarche status, menopausal symptoms, and so on. they were followed up for 5 years. During the non-epidemic period, patients were usually followed up in the clinic every 6 months. During the outbreak, follow-up assessments were performed *via* telephone. All participants provided written informed consent before. This study was approved by the Medical Ethics Committee of Peking University People's Hospital and the Medical Ethics Committee of Hebei Yanda Ludaopei Hospital (NO. 2020PHB017-01).

### 2.2. Patients

Patients, less than age 40 at the time of HCT for blood disease were included in the study. Exclusion criteria included the following: (1) presence of POI, premature ovarian failure, or sexual development abnormalities before treatment (0 patient); (2) history of ovarian surgery (0 patient); (3) receipt of second transplantation (2 patients).

### 2.3. Data variables

An independently designed questionnaire was adapted to gather the following information: general information (age, height, weight), information on blood disorders [type of hematologic disease, lines of chemotherapy before transplantation, donor type, conditioning regimen, acute graft-vs.-host disease (aGVHD) grade], and information related to gynecology (menstrual status before and after HCT, parity status, awareness of the protection of reproductive function, and hormone replacement therapy) were collected. The modified Kupperman menopausal index (KMI) was used to evaluate the severity of the menopausal symptoms. Data were collected by telephone or network questionnaire and analyzed by gynecologists or hematologists about 3 years after transplantation.

#### 2.3.1. Definitions of variables

Premature ovarian insufficiency was defined as a clinical condition in postmenarchal women <40 years of age and characterized by the absence of menstrual cycles (amenorrhea) for ≥4 months and 2 elevated serum follicle-stimulating hormones (FSH) levels in the menopausal range, or delayed or arrested pubertal progression in girls ≥ 13 years.

Menopausal symptoms were determined by the modified Kupperman Index and classified as none (total score <6), mild (6 ≤ total score ≤ 15), or moderate (16 ≤ total score ≤ 30), and severe (total score >30). Please refer to the literature (30).

### 2.4. Statistics

All statistical analyses were performed using a two-tailed test with a value of *p* < 0.05 being considered statistically significant. Data are presented as mean ± SD. The Chi-square test and Fisher's exact test were used for significance analyses of categorical variables and Fisher's exact test was used to compare results with significant differences. IBM SPSS software v20.0 was used for all statistical analyses.

## 3. Results

### 3.1. Baseline characteristics of the patients

**Figure 1:**
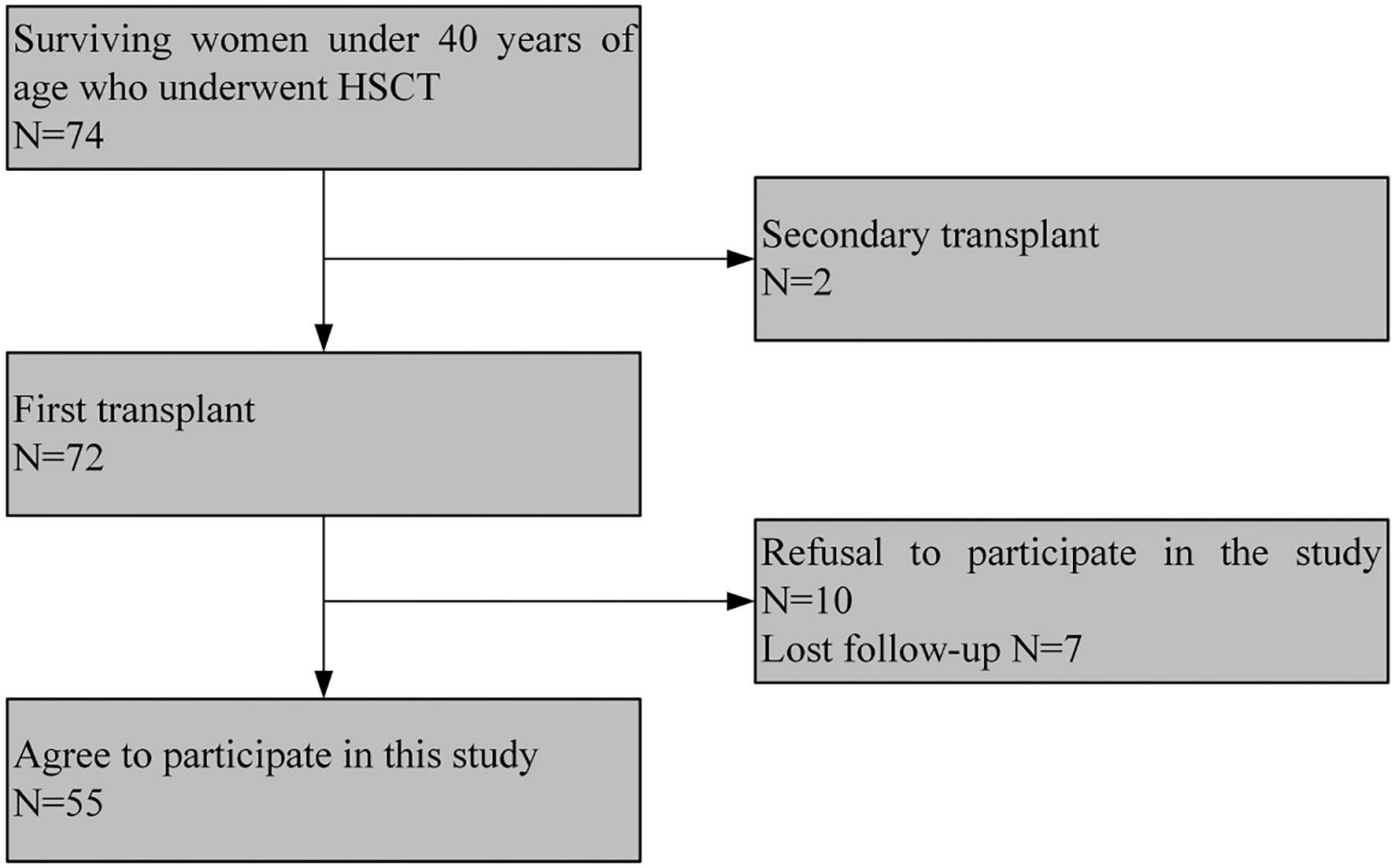
Consort diagram of patients.

Of the total of 74 patients under the age of 40 who underwent allo-HCT at Hebei Yanda Ludaopei Hospital, 2 women did not meet the inclusion criteria: 2 cases received secondary transplants. Among the remaining 72 cases, 10 women refused to participate in the study, and 7 cases lost follow-up. Eventually, 55 cases completed the survey. The flowchart is shown in Figure 1.

#### 3.1.1. Baseline characteristics

Among the 55 cases, the average age of transplantation was 20.45 years (ranging from 8 to 37), so various participants of childhood, adolescence, and childbearing ages were involved.

Malignant hematological diseases were chiefly represented by acute lymphoblastic leukemia (ALL) and acute myeloid leukemia (AML) (80%). Patients with AA accounted for only 9.1% and the remaining 10.9% of patients suffered from hypocellular leukemia, lymphoma, chronic active EBV infection, and lymphoid blast phase of chronic myeloid leukemia. All patients, except those with aplastic anemia (AA), were treated with a myeloablative conditioning regimen (MAC) (90.9%), of whom 45.5% received the modified total body irradiation (TBI)/ cyclophosphamide (Cy) regimen and 41.8% the modified busulfan (Bu)/Cy regimen. cGVHD was present in 50.9% of patients. Baseline characteristics of the study cohort are given in Table 1.

**Table 1:** Baseline characteristics of the patients (*N* = 55).

**Characteristics (*n* = 55)**	**Number (*%*)**
**Age of HCT (year)**	
≤ 10	6 (10.9)
11–15	16 (29.1)
16–20	7 (12.7)
21–40	26 (47.3)
**Disease type**	
ALL	21 (38.2)
AML	23 (41.8)
AA	5 (9.1)
Other	6 (10.9)
**Lines of chemotherapy (not including conditioning regimen)**	
0	6 (10.9)
≤ 5	24 (43.6)
5–10	15 (27.3)
>10	10 (18.2)
**Type of allogeneic HCT**	
Haploidentical	38 (69.1)
Unrelated	10 (18.2)
Matched sibling	7 (12.7)
**HLA match**	
5/10	23 (41.8)
6/10-9/10	19 (34.5)
10/10	13 (23.6)
**Conditioning regimen**	
MAC	51 (92.7)
TBI/Cy	25 (45.5)
Bu/Cy	23 (41.8)
Flu+Ara-C/Cy	3 (4.3)
RIC	4 (7.3)
**aGVHD grade**	
0	31 (56.4)
I	11 (20.0)
II	8 (14.5)
III	3 (5.5)
IV	2 (3.6)
**cGVHD**	
No	27 (49.1)
Yes	28 (50.9)
**Relapse**	
No	52 (94.5)
Yes	3 (5.5)

ALL, acute lymphoblastic leukemia; AML, acute myeloid leukemia; AA, aplastic anemia; HLA, human leukocyte antigen; MAC, myeloablative conditioning regimen; TBI, total body irradiation; Cy, cyclophosphamide; Bu, busulfan; Flu, fludarabine; Ara-C, cytosine arabinoside; RIC, reduced-intensity conditioning; aGVHD, acute graft-vs.-host disease.

### 3.2. The menstruation and menopausal symptoms of patients before and after HCT

As shown in Table 2, before transplantation, 13/55 (23.6%) had no menarche, 33/55 (60%) had relatively regular menstrual cycles (menstrual cycle ≤60 days), and 9/55 (16.4%) had irregular menstrual cycles (menstrual cycle>60 days). Only 5/55 (9.1%) patients said they were aware of pre-transplantation reproductive and ovarian protection treatments, such as egg-freezing, ovarian cryopreservation, and GnRH-a injection. No patient received fertility protection. Among women without pre-transplantation menarche, 8/13 (61.5%) later experienced spontaneous menarche; 7/42 (16.67%) had a spontaneous menstrual relapse and 35/42 (83.3%) had amenorrhea after transplantation. Only 1/55 (1.8%) had severe menopausal symptoms.

**Table 2:** The menstruation and menopausal symptoms of patients before and after HCT.

**Symptoms (*n* = 55)**	**Age**	**Number (*%*)**
**Menstruation before transplantation**		
No menarche	12 ± 5.7	13 (23.6)
Regular	22.5 ± 20.5	33 (60.0)
Irregular	24 ± 18.4	9 (16.4)
**Sexually active before transplantation**		
Yes	31 ± 8.4	21 (38.2)
No	12.5 ± 6.3	34 (61.8)
**Given birth before transplantation**		
Yes	34 ± 4.2	18 (32.73)
No	16.5 ± 0.7	37 (67.27)
**Aware of the protection of**		
**reproductive function before transplantation**		
Yes	22.5 ± 20.5	5 (9.1)
No	21.0 ± 12.7	50 (90.9)
**Reproductive function protection before transplantation**		0 (0)
**Menstruation after transplantation**		
No menarche		5 (9.1)
Spontaneous menarche		8 (14.5)
Spontaneous menstrual relapse		7 (12.7)
Amenorrhea		35 (63.6)
**Sexually active after transplantation**		
Yes	29.0 ± 2.1	12 (21.8)
No	19.0 ± 2.8	36 (78.2)
**Pregnancy after transplantation**		0 (0)
**Whether to diagnosed with POI**		
Yes	18.5 ± 3.5	40 (72.7)
No	12.5 ± 6.4	15 (27.3)
**Kupperman index after transplantation**		10 ± 8.41
<6 (Asymptomatic)	12.0 ± 5.7	24 (43.6)
6–15 (Mild)	11.5 ± 0.7	16 (29.1)
16–30 (Moderate)	12.5 ± 2.1	14 (25.5)
>30 (Severe)	32.0 ± 0.0	1 (1.8)
**Hormone replacement therapy**		
Yes	9.5 ± 2.1	22 (40.0)
No	22.0 ± 14.1	33 (60.0)

### 3.3. Correlations between the incidence of POI/menopausal symptoms and different clinical factors

As shown in Table 3, age at transplantation (*p* < 0.001), conditioning regimen with TBI/Cy (*p* < 0.001), conditioning regimen with Bu/Cy regimen (*p* = 0.001) and AML (*p* < 0.01) are factors affecting POI. Pairwise comparisons revealed that the probability of POI in patients aged ≤10 years at transplantation (0%) was significantly lower than that in patients aged 11–20 (62.5%; *p* < 0.001) or 21–40 (100%; *p* < 0.001). For the 6 patients aged ≤10 years at transplantation, the median age was 9 years (range, 8–10 years). They all had spontaneous menarche or spontaneous menstrual relapse. The probability of POI in patients aged 11–15 years was 56.3%, and the probability of POI in patients aged 16–20 years was 71.4%. For the 5 patients who had no menarche, the median age was 12.2 years (range, 11–16 years) and all of them had serum FSH at menopausal level (>40 IU/l). The probability of POI in patients aged 11–20 years was significantly lower than in patients aged 21–40 (*p* < 0.01). The probability of POI in patients receiving the TBI/Cy regimen (50%) was significantly lower than in those receiving chemotherapy alone (96.3%; *p* < 0.001). The probability of POI in patients receiving the Bu/Cy conditioning regimen (96%) was significantly higher than in those receiving other regimens (53.3%; *p* < 0.001). In addition, patients with AML were more likely to have POI (95.7%; *p* < 0.01). Kupperman scores were significantly correlated with age at transplantation. Although no significant difference was shown by pairwise comparison, the occurrence of moderate and severe menopausal-related symptoms (Kupperman score > 15) was lower in patients aged ≤10 (0%) or 11-20 years (16.7%) than in those of 21-40 years. Among older patients, 44% had Kupperman scores of >15. In addition, HLA matching, aGVHD grade, and cyclosporine use were also analyzed but none showed a correlation with POI diagnosis or Kupperman score. One-way correlation analysis of the decision to accept HRT and the Kupperman score revealed no significant difference.

**Table 3:** Correlations between the incidence of POI/menopausal symptoms and different clinical factors.

	**POI**			**Kupperman score** >**15**		
	**Number**	**Proportion (*%*)**	***P***	**Number**	**Proportion (*%*)**	***P***
**Age at transplantation**						
≤ 10	0	0	<0.001	0	0	0.028
11-20	15	62.5		4	16.7	
21-40	25	100		11	44	
**Lines of chemotherapy**						
0	5	83.3	NS	0	0	NS
≤ 5	19	79.2		8	33.3	
6-10	12	80		5	33.3	
>10	4	40		2	20	
**Type of disease**						
AML	22	95.7	<0.01	8	34.8	NS
ALL	11	52.4		7	33.3	
AA	4	80		0	0	
Other	3	50		0	0	
**Type of transplantation**						
Matched Sibling	6	85.7	NS	2	28.6	NS
Haploidentical	26	68.4		11	28.9	
Unrelated	8	80		2	20	
**TBI/Cy**						
No	26	96.3	<0.001	8	29.6	NS
Yes	14	50		7	25	
**Bu/Cy**						
No	16	53.3	0.001	8	26.7	NS
Yes	24	96		7	28	
**cGVHD**						
No	17	63	NS	7	25.9	NS
Yes	23	82.1		8	28.6	
**Menarche at treatment**						
pre menarche	13	38.5	0.003	1	7.7	NS
post menarche	42	83.3		14	33.3	

### 3.4. Factors affecting POI in children and adolescent females

Since the post-HCT incidence of POI in women aged 21–40 years reached 100%, a stratified analysis of factors affecting POI in children and adolescents of ≤ 20 years was conducted. POI incidence (6.7%) in patients receiving the TBI/Cy regimen was significantly lower (*p* < 0.001). In addition, the POI incidence of (92.3%) in those receiving the Bu/Cy conditioning regimen was significantly higher (*p* < 0.001). Patients with AML were more likely to have POI (90.9%; *p* < 0.001). Patients with transplantation performed after menarche are more likely to appear with POI (83.3%; *p* = 0.003).

## 4. Discussion

Hematopoietic stem cell transplantation involves the elimination of abnormal hematopoietic cells through conditioning regimens, such as radiotherapy and chemotherapy, followed by transplantation of donor or autologous hematopoietic stem cells to replenish the hematopoietic and immune systems. Worldwide, the number of HCTs has shown sustained growth for decades. With the annual global frequency of HCT increasing and prolonged post-HCT survival times, protection of reproductive and ovarian function becomes increasingly important. The last report from the Chinese Blood and Marrow Transplantation Registry Group (CBMTRG) described a continued growth of transplant activity in China (8).In 2019, the total number of HCTs in China reached more than 10,000 for the first time, to date, the reproductive and ovarian function of post-HCT Chinese has received little attention. The current study aimed to address this deficit.

Most patients enrolled in the current study suffered from ALL or AML (80%) and their ages at the time of transplantation ranged from 8-37 years. Except for those patients who had anemic hematological diseases, all were treated with myeloablative pre-HCT conditioning regimes, such as modified TBI/Cy and Bu/Cy programs. Such programs are compatible with various individualized medications, including Me-CCNU, idarubicin (IDA), antithymocyte globulin (ATG), and fludarabine (FLU). The present study is restricted to a consideration of TBI and alkylating agents which have the greatest impact on the ovary. The current study is a comprehensive description of pregnancy, menstruation, and menopause-related symptoms of post-HCT Chinese patients 3 years after transplantation. The number of women who were sexually active following HCT (21.8%) was fewer than before transplantation (38.2%) and the pregnancy rate was 0. Patients generally are advised to avoid pregnancy from the time of pretransplant evaluation through at least 2 years (malignant blood disease) or 3-5 years (benign blood disease) post-HCT and often longer because of the risk of relapse and continued use of potentially teratogenic, transplant-related medications, although this recommendation is tailored for individual patients (31). Sanders et al. (13) have reported a 4.5% pregnancy rate in post-pubertal women following transplantation. Vatanen et al. (32) evaluated the ovarian function among 92 adult or pubertal female survivors during 1978–2000, at a mean age of 9±4.3 years (range 1–19). Ten women out of the 92 survivors had a total of 14 pregnancies and gave birth to 12 children. The current study reveals a very low awareness rate (9.1%) regarding protective treatment for reproductive function among women receiving HCT and none had undergone such treatment. In 2017, local doctors were not aware that transplants could cause POI, were unaware of alternative fertility protection methods, and did not inform them of the risks in advance. At that time, this situation was also widespread in other hospitals in China, but it has improved significantly now, and we have published relevant consensuses (33). Such findings indicate the crucial nature of appropriate cooperation between departments of hematology and obstetrics and gynecology for the future. Hematologists should fully inform the patient before transplantation and make a referral to the obstetrician and reproductive specialist for consultation and treatment. Post-HCT ovarian function was evaluated, revealing that 72.7% (40) of participants in the current study could be reliably diagnosed with POI. Of 13 recipients of treatment during childhood who had no menarche before transplantation, 8 experienced spontaneous menarche, as did 7 women who did have menarche before transplantation. All women aged >20 years at the time of transplantation had amenorrhea. These observations suggest that a minority of women retain ovarian function after HCT. Whereas some young women experience post-HCT menstrual cramps, hormone tests show values for FSH > 40U/L, indicating that POI has developed. In women > 20 years old, the probability of POI is 100%. The current study included one subject who was 20 at the time of transplantation and had a voluntary menstrual blast for 4 years post-transplantation, resulting in FSH = 6U/L and recovery of ovarian function. However, further observations are required to demonstrate full recovery of ovarian function. Menopausal-related symptoms experienced post-transplantation were relatively mild, perhaps due to the younger ages of enrolled patients. Only 40% of patients received post-operative HRT. We believe that HRT should always be given in the post-transplantation period, even to women with spontaneous menarche or relapse, to achieve the therapeutic effect of inducing puberty and primary prevention (34). Consistent with the results of previous studies, age is the most important factor influencing the incidence of POI: the younger the age at transplantation, the lower the risk of POI. POI incidence among those of 21–40 years was 100%; 62.5% for those aged 11–20 and 0% for those aged ≤10 years (32). Pascale et al. (24) suggested that the younger group showed clinical evidence of ovarian function after BMT significantly more often than the older group (71 vs. 22%; *P* < 0.01). Logistic regression analysis confirmed an independent protective effect of young age at the time of BMT (*P* = 0.004). These findings are likely to be related to the gradual atresia of ovarian follicles after birth. In addition, this study finds that women receiving TBI/Cy are less susceptible to developing POI than those receiving chemotherapy alone. This result is inconsistent with previous research works carried out by Jadoul et al. (24) and Vatanen et al. (27), and their detailed results can be found in the corresponding literature cited here. Differences in radiotherapy and chemotherapy regimens in different centers may account for these discrepancies. TBI administered by the center scrutinized in the current study adopted accelerated hyperfractionated radiation therapy. Following 3 days of irradiation, there was no special protection for the pelvic cavity. Such an approach may be less damaging to the ovaries. By contrast, the home Bu/Cy regimen involves a relatively large dose of intravenous medication for 4 days with no liver first-pass effect. The probability of POI in patients with AML (95.7%) was higher than that in patients with ALL (52.4%) or patients with AA (80%), perhaps due to variations in radiotherapy and chemotherapy dosage. Stratified analysis was conducted to clarify factors related to POI onset among women of <20 years at transplantation. The TBI/Cy regimen was associated with low POI incidence and the Bu/Cy regimen with high incidence. To the best of our knowledge, the current retrospective prospective study is the first to report a complete analysis of post-HCT reproductive and ovarian function in Chinese women. A novel finding is that the use of linear accelerators and hypersegmentation schemes during the TBI/Cy conditioning regimen program was less damaging to the ovaries. We acknowledge some shortcomings in our research. The sample size was small, confounding factors could not be satisfactorily controlled and multivariate analysis could not be performed. Moreover, the follow-up time was short. However, we believe that there is an urgent need to improve the protection of ovarian function before transplantation and to provide HRT treatment after transplantation to bring about increased survival rates and quality of life for patients with HCT.

## 5. Conclusion

Premature ovarian insufficiency incidence in women after HCT is 72.7%, including rates of 100% for transplant recipients aged 21–40, 62.5% for those aged 11–20, and 0% for those ≤10 years old. Protective factors for the development of post-HCT POI include young age at transplantation and a modified TBI/Cy conditioning regimen. Risk factor includes the Bu/Cy conditioning regimen program and AML. Symptoms related to menopause were related to the age at transplantation with younger women having lower Kupperman scores. Children, adolescents, and young women with POI should be managed by a multidisciplinary team including gynecologists, pediatricians, endocrinologists, dietitians, and psychologists.

## Data availability statement

The raw data supporting the conclusions of this article will be made available by the authors, without undue reservation.

## Ethics statement

This study was approved by the Medical Ethics Committee of Peking University People's Hospital (Project no.2018PHB085-01). Written informed consent to participate in this study was provided by the participants' legal guardian/next of kin.

## Author contributions

HS and XZ wrote the article. HS wrote ethical materials and a cooperation agreement. YZ and XY conceived and designed the experiments. HS, XZ, and YZ collected the data and analyzed experimental data. YL, DL, and JZ revised the manuscript critically during the revision stage. All authors contributed to the article and approved the submitted version.

## Funding

The study was supported by Roche Diagnostics: Dynamic study for the effects of chemotherapy and bone marrow transplantation on ovarian function in pre-adolescent hematological disease survivors (Project no. 2018PHB085-01).

## Conflict of interest

The authors declare that the research was conducted in the absence of any commercial or financial relationships that could be construed as a potential conflict of interest.

## Publisher's note

All claims expressed in this article are solely those of the authors and do not necessarily represent those of their affiliated organizations, or those of the publisher, the editors and the reviewers. Any product that may be evaluated in this article, or claim that may be made by its manufacturer, is not guaranteed or endorsed by the publisher.
